# Primary Intratesticular glomus tumor: a rare mesenchymal neoplasm and a diagnostic challenge

**DOI:** 10.1093/omcr/omag075

**Published:** 2026-10-04

**Authors:** Zahra Aminparast, Payam Nikjo, Donya Rahmati, Paria Nikjo

**Affiliations:** Department of Pathology, Kermanshah University of Medical Sciences, Imam Reza Hospital Complex, Parastar Boulevard, Kermanshah, Kermanshah Province, 6714415333, Iran; Department of Pathology, Kermanshah University of Medical Sciences, Imam Reza Hospital Complex, Parastar Boulevard, Kermanshah, Kermanshah Province, 6714415333, Iran; Department of Pathology, Kermanshah University of Medical Sciences, Imam Reza Hospital Complex, Parastar Boulevard, Kermanshah, Kermanshah Province, 6714415333, Iran; Department of Pathology, School of Medical Sciences, Islamic Azad University, Basij Street, Ardabil, Ardabil Province, 5615731567, Iran

**Keywords:** glomus tumor, testis, Intratesticular mass, mesenchymal neoplasm, immunohistochemistry

## Abstract

Background: Glomus tumors are rare mesenchymal neoplasms of perivascular origin, most commonly found in the dermis or subcutis of distal extremities. Testicular involvement is exceptionally uncommon and poses a diagnostic challenge due to histopathologic similarities with other intratesticular tumors. Case Presentation: We describe an 85-year-old man with a painless, gradually enlarging left testicular mass. Imaging and tumor markers were inconclusive. Radical orchiectomy revealed a well-circumscribed intratesticular lesion. Histology showed uniform round cells with eosinophilic cytoplasm arranged around vascular spaces. Immunohistochemistry demonstrated strong positivity for SMA and negativity for germ cell, stromal, neuroendocrine, and melanocytic markers, confirming the diagnosis of a primary intratesticular glomus tumor. Conclusion: This case underscores the need to consider rare mesenchymal tumors in the differential diagnosis of testicular masses in elderly patients and highlights the pivotal role of immunohistochemistry in establishing an accurate diagnosis.

## Introduction

Glomus tumors are rare, usually benign perivascular mesenchymal neoplasms originating from modified smooth muscle cells of the glomus body. They predominantly occur as painful subungual nodules in the fingers and toes, favoring distal extremities [1]. Involvement of visceral organs or deep soft tissues is exceptionally uncommon, and testicular localization is exceedingly rare, with only a few cases reported in the literature [2]. Due to overlapping histopathological features with more common testicular neoplasms—particularly germ cell tumors and sex cord-stromal tumors—accurate diagnosis demands a high index of suspicion and a thorough immunohistochemical (IHC) evaluation [3]. Misdiagnosis can result in inappropriate management or overtreatment. This report presents a primary intratesticular glomus tumor in an elderly patient and highlights its clinical, histopathological, and immunohistochemical characteristics, along with a discussion of the differential diagnosis and relevant literature.

## Case presentation

An 85-year-old man presented with a five-month history of a painless, gradually enlarging mass in his left testis. He denied trauma, systemic illness, prior malignancy, or long-term medication use.

On examination, the left testis was enlarged, firm, and non-tender, without erythema or scrotal skin involvement. No inguinal lymphadenopathy was detected. Laboratory investigations, including complete blood count, liver and renal function tests, and inflammatory markers (ESR, CRP), were within normal limits. Serum tumor markers—AFP (4.6 ng/ml), β-hCG (<1.0 mIU/ml), and LDH (148 U/l)—were unremarkable.

Scrotal ultrasonography revealed a well-circumscribed, hypoechoic lesion measuring 1.8 × 1.2 cm at the lower pole of the left testis, with mild internal vascularity on Doppler imaging. No calcifications, cystic areas, hydrocele, or varicocele were observed. The surrounding parenchyma appeared normal.

The patient underwent radical left orchiectomy. Gross examination showed a gray testis measuring 5 × 4 × 1.8 cm, with a well-demarcated, creamy-white mass of 1.7 cm at the lower pole (Fig. 1).

**Figure 1:**
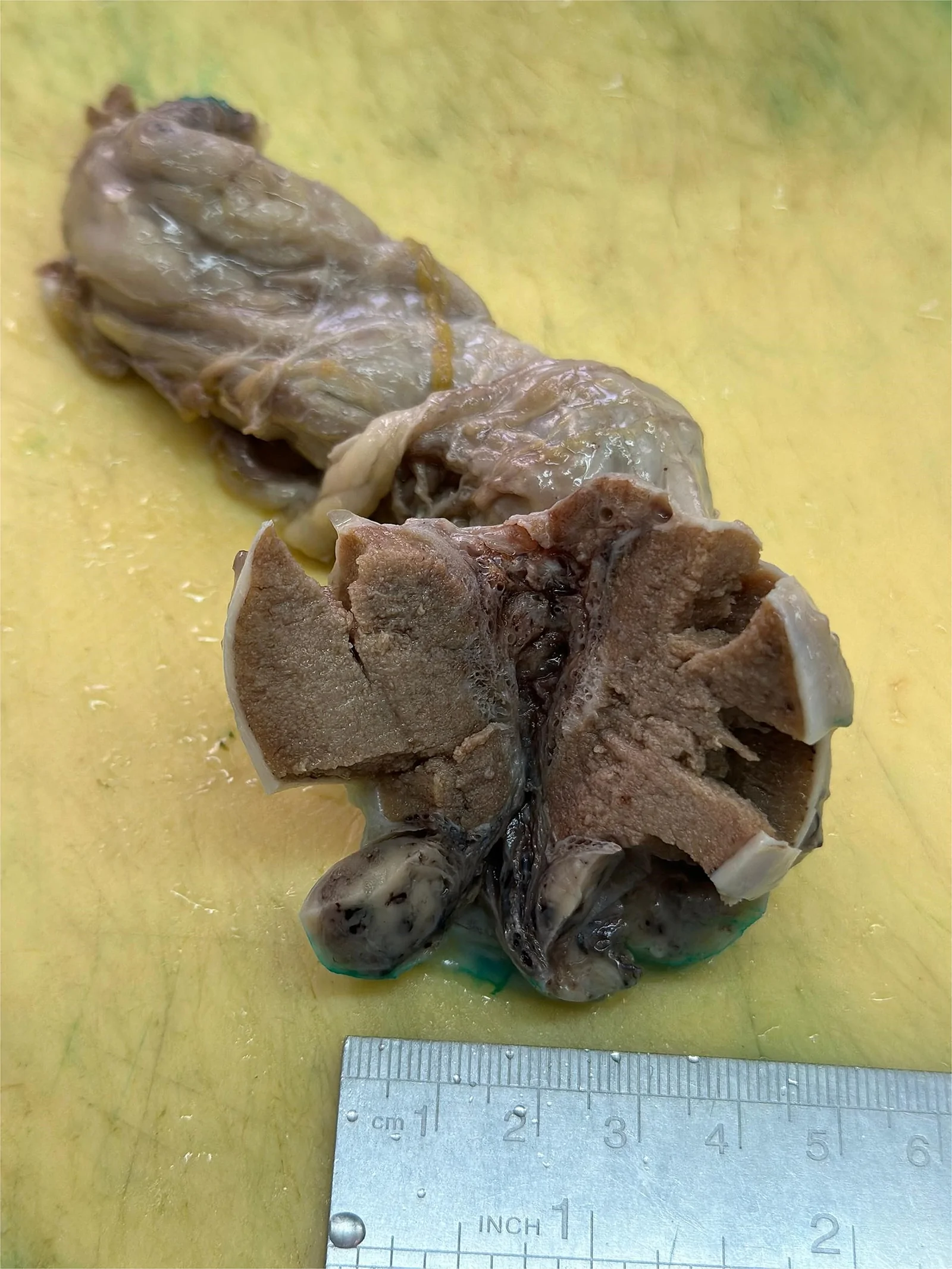
A well-circumscribed, creamy-colored mass measuring 1.7 cm is identified at the lower pole of the testis, photo by author.

Histology demonstrated a sharply circumscribed neoplasm composed of uniform round-to-oval cells with pale eosinophilic cytoplasm and centrally placed nuclei, arranged in a prominent perivascular pattern. No features of germ cell, sex cord-stromal, or metastatic tumors were identified. Mitotic figures were rare, necrosis was absent, and margins were tumor-free (Fig. 2).

**Figure 2:**
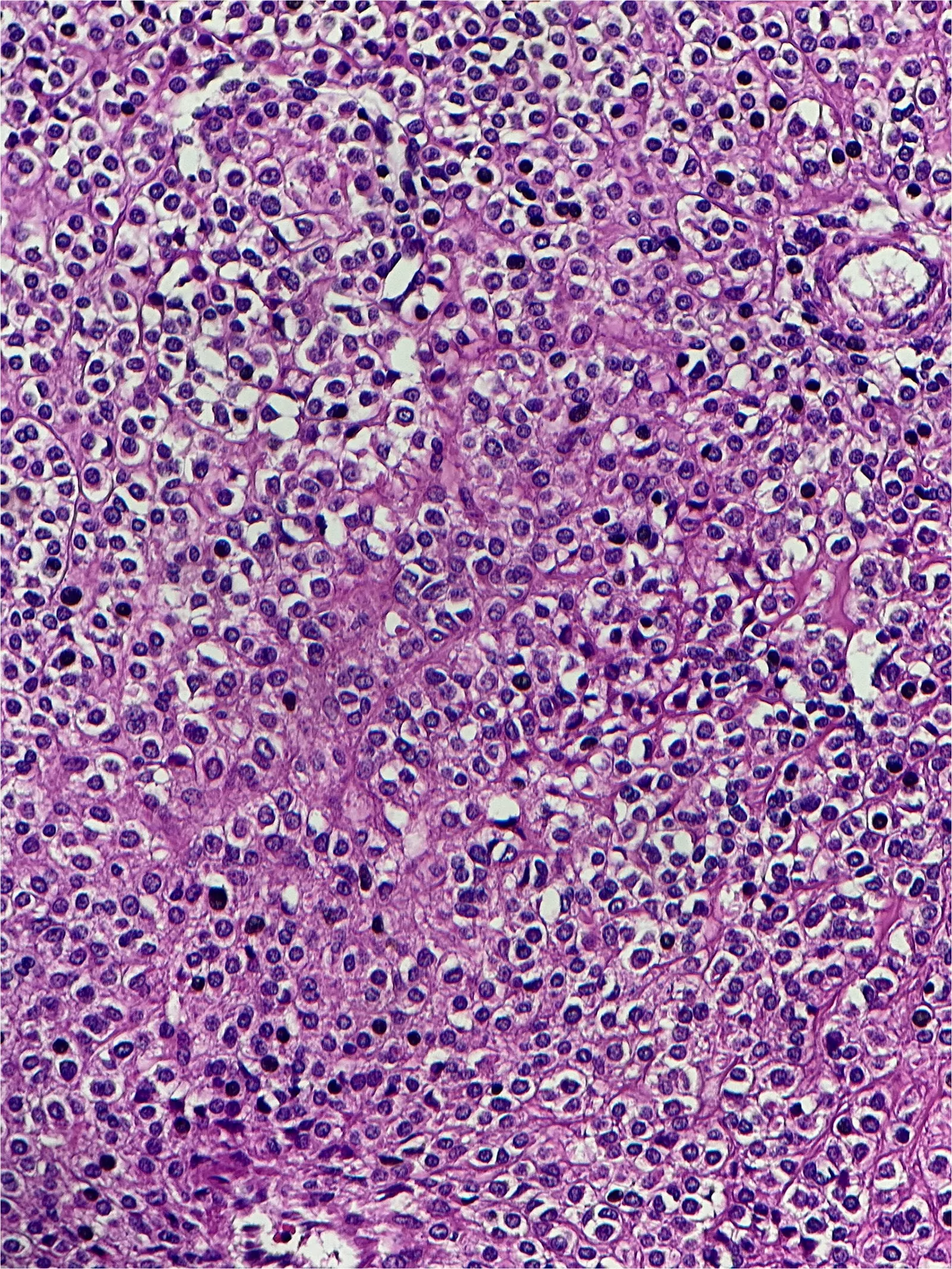
H&E staining demonstrates glomus tumor,composed of uniform round to oval cells with pale eosinophilic cytoplasm and centrally located nuclei arranged in a perivascular pattern (×20) photo by author.

Immunohistochemistry revealed diffuse cytoplasmic positivity for SMA (Fig. 3), with CD34 staining limited to endothelial cells and faint BCL2 expression. The tumor was negative for germ cell (PLAP, OCT3/4, SALL4, CD30), sex cord-stromal (Inhibin, Calretinin), melanocytic (S100, HMB-45), neuroendocrine (Synaptophysin, Chromogranin), lymphoid (CD45), and epithelial markers (CK, EMA), confirming a primary intratesticular glomus tumor.

**Figure 3:**
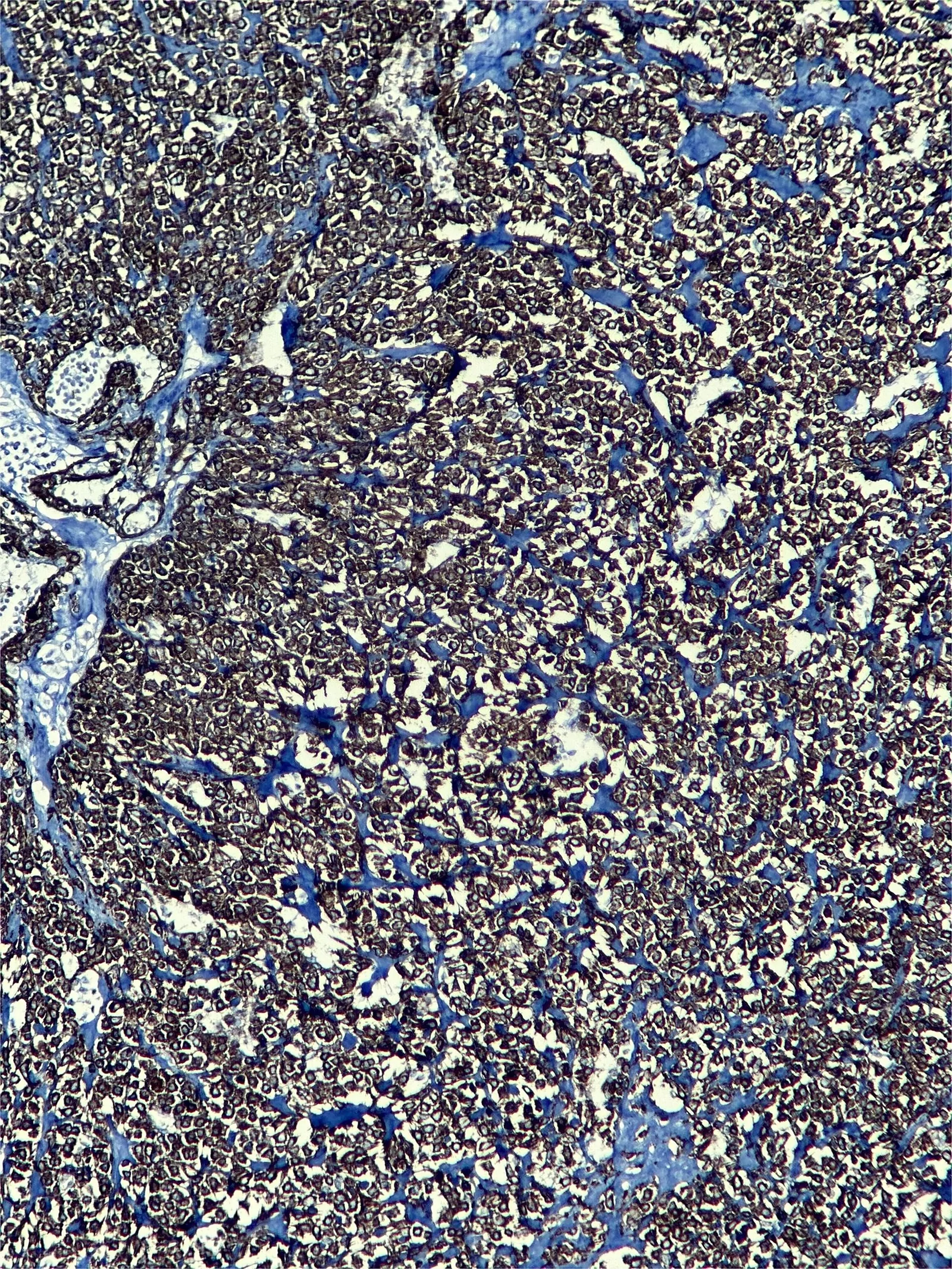
Immunohistochemical staining was positive for SMA (×20) photo by. author.

Molecular analysis (e.g. NOTCH mutations) was not performed due to resource limitations. At three-month follow-up, the patient remained asymptomatic with no evidence of recurrence or metastasis.

## Discussion

Glomus tumors are rare perivascular mesenchymal neoplasms, representing less than 2% of soft tissue tumors. They most commonly arise in the dermis or subcutis of distal extremities, particularly in the subungual region [4]. Visceral involvement is exceedingly rare, and primary intratesticular glomus tumors are an exceptionally uncommon entity, with very few definitive cases reported [2].

Our case highlights a glomus tumor entirely confined to the testicular parenchyma, without paratesticular involvement. This localization is diagnostically challenging because most intratesticular masses in elderly patients are germ cell tumors or lymphomas. Misclassification may result in overtreatment, including unnecessary chemotherapy or extensive surgery, which are not indicated for benign mesenchymal lesions.

Radiologic evaluation was non-specific; scrotal ultrasonography may show hypoechoic or heterogeneous masses with prominent vascularity, mimicking other hypervascular tumors such as Leydig cell tumors or seminomas. Therefore, imaging alone is insufficient for diagnosis, and histopathology combined with immunohistochemistry (IHC) is essential [5].

Histologically, glomus tumors are composed of uniform round-to-oval cells with pale eosinophilic cytoplasm and centrally located nuclei, often arranged in a perivascular pattern [1]. These features, although distinctive, can overlap with other small round cell tumors of the testis including seminoma, lymphoma, neuroendocrine tumors, and sex cord-stromal tumors [6]. The differential diagnosis is particularly challenging in small biopsies or limited specimens [7].

Immunohistochemistry is pivotal in establishing the diagnosis. Diffuse SMA positivity, combined with negativity for germ cell (PLAP, OCT3/4, SALL4, CD30), sex cord-stromal (Inhibin, Calretinin), melanocytic (S100, HMB-45), neuroendocrine (Chromogranin, Synaptophysin), and lymphoid markers (CD45), confirms a glomus tumor. CD34 positivity is restricted to endothelial cells, and faint BCL2 expression may be observed. Table 1 summarizes key histologic and immunophenotypic differences between intratesticular glomus tumors and their main differential diagnoses [8].

**Table 1:** Differential diagnosis of intratesticular glomus tumor and its key histologic and immunohistochemical distinctions.

Feature	Glomus Tumor	Seminoma	Leydig Cell Tumor	Testicular Lymphoma
Typical Age	Middle-aged to elderly adults	20–45 years	20–60 years	>60 years
Clinical Presentation	Painless, slowly growing mass	Painless testicular enlargement	Testicular mass ± gynecomastia	Painful or painless mass, often bilateral in older men
Gross Appearance	Small, firm, well-circumscribed cream-colored mass	Homogeneous, gray-white, no necrosis	Yellow-brown, homogeneous, ± hemorrhage	Gray-white, infiltrative with tunica/albuginea involvement
Microscopic Features	Uniform round cells with eosinophilic cytoplasm, perivascular pattern	Large cells with clear cytoplasm, central nuclei, fibrous septa with lymphocytes	Large eosinophilic cells, Reinke crystals in ~ 30%	Sheets of atypical lymphoid cells with high mitotic activity
SMA	Positive (diffuse)	Negative	Negative	Negative
PLAP	Negative	Positive	Negative	Negative
OCT3/4	Negative	Positive	Negative	Negative
Inhibin	Negative	Negative	Positive	Negative
Calretinin	Negative	Negative	Positive (variable)	Negative
CD45	Negative	Negative	Negative	Positive
CD34	Positive only in vessel walls	Negative	Negative	Negative
S100/HMB45	Negative	Negative	Negative	Negative
Ki-67 Proliferation Index	Low (<5%)	Moderate to high	Usually low	High (30–90%)

Note: Glomus tumors show diffuse SMA positivity and lack expression of germ cell, stromal, melanocytic, lymphoid, and neuroendocrine markers. Seminomas typically express PLAP, OCT3/4, and SALL4. Leydig cell tumors are positive for Inhibin, Calretinin, and occasionally Melan-A. Testicular lymphomas express CD45 and lineage-specific markers such as CD20 (B-cell) or CD3 (T-cell).

From a clinical perspective, recognizing this entity is particularly important in elderly patients, in whom intratesticular masses are often presumed malignant. In selected cases, awareness of this diagnosis may support consideration of testis-sparing approaches when clinicoradiologic and intraoperative findings are reassuring.

Although glomus tumors are generally benign, visceral or atypical cases may harbor NOTCH1/2 mutations, which could contribute to tumorigenesis and risk stratification [9]. Molecular testing was not performed in our case due to resource limitations. Complete surgical excision with negative margins is curative, and recurrence is rare [10]. Malignant transformation is extremely uncommon and is associated with large size (>2 cm), deep location, cytologic atypia, necrosis, or high mitotic rate (>5/50 HPF) [11]. None of these features were observed in our patient.

In conclusion, primary intratesticular glomus tumors, although rare, should be considered in the differential diagnosis of testicular masses in elderly patients. Careful histopathological assessment and judicious use of IHC are critical to avoid misdiagnosis and unnecessary treatment.

## Conclusion

This case illustrates a rare occurrence of a primary intratesticular glomus tumor, a neoplasm that presents significant diagnostic challenges due to its histologic overlap with more common testicular tumors. Accurate diagnosis requires careful histopathological evaluation and judicious use of immunohistochemistry to exclude malignant differentials. Recognition of such rare benign mesenchymal tumors is essential to avoid unnecessary aggressive treatment, particularly in elderly patients. This case underscores the importance of including rare benign mesenchymal tumors in the differential diagnosis of intratesticular masses to avoid overtreatment.

## Supplementary Material

Supplementary_File_omag075
