# Supplementary material for: Primary Intratesticular glomus tumor: a rare mesenchymal neoplasm and a diagnostic challenge
Source: Oxf Med Case Reports. 2026 Oct 4;2026(10):omag075. doi: 10.1093/omcr/omag075 (PMC13677788; doi:10.1093/omcr/omag075)
Supplement: Supplementary file 1 [file supplementary_file_omag075.docx]

Fig. 1: A well-circumscribed, creamy-colored mass measuring 1.7 cm is identified at the lower pole of the testis, photo by author.

Fig. 2: H&E staining demonstrates Glomus tumor ,composed of uniform round to oval cells with pale eosinophilic cytoplasm and centrally located nuclei arranged in a perivascular pattern ( ×20) photo by author.

Fig. 3: : Immunohistochemical staining was positive for SMA ( ×20) photo by

author.
